# Impact of nitrogen flushing and oil choice on the progression of lipid oxidation in unwashed fried sliced potato crisps

**DOI:** 10.1016/j.foodchem.2015.11.136

**Published:** 2016-05-15

**Authors:** E. Marasca, D. Greetham, S.D. Herring, I.D. Fisk

**Affiliations:** a School of Biosciences, University of Nottingham, Loughborough, Leicestershire LE12 5RD, UK; b Pipers Crisps Ltd, Wellington House, Lincolnshire DN20 0SP, UK

**Keywords:** Crisps, Sunflower oil, High oleic sunflower oil, Shelf-life tests, Oxidation products

## Abstract

Unwashed, sliced, batch-fried potato crisps have a unique texture and are growing in popularity in the UK/EU premium snack food market. In this study, the storage stability of unwashed sliced (high surface starch) potatoes (crisps) fried in regular sunflower oil (SO) or in high oleic sunflower oil (HOSO) was compared over accelerated shelf life testing (45 °C, 6 weeks); with and without nitrogen gas flushing. Primary oxidation products (lipid hydroperoxides) were measured with a ferrous oxidation-xylenol orange (FOX) assay and volatile secondary oxidation products (hexanal) were quantified by using solid phase micro-extraction gas chromatography mass spectrometry (HS-SPME-GC/MS). Results revealed that crisps fried in SO were the least stable. Flushing the stored crisps with nitrogen gas proved to be effective in slowing down the oxidation rate after frying with sunflower oil, significantly stabilizing the crisps. However, crisps fried in HOSO were the most stable, with the lowest rate of development of oxidation markers, and this has previously not been shown for crisps with a high free starch content.

## Highlights

• Storage stability of unwashed potato crisps fried in sunflower oil’s was compared.
• Oxidation products were determined, with and without flushing with nitrogen gas.
• Crisps fried in SO were the least stable and those using HOSO the most stable.
• Presence of nitrogen gas slowed down the oxidation rate after frying.

## 1 Introduction

Rancidity of edible oils and fatty foods due to lipid oxidation is a serious problem within the deep-frying industry (Gómez-Alonso et al., 2004, Matthäus, 2007, Sanches Silva et al., 2004, Sanches-Silva et al., 2004). Autoxidation, defined as the spontaneous reaction of atmospheric oxygen with lipids (St Angelo, 1996), is the most common process leading to oxidative deterioration and, as a consequence, to rancidity. Lipid hydroperoxides are the primary products of autoxidation and decomposition of the hydroperoxides leads to the formation of aldehydes, ketones, alcohols, hydrocarbons, volatile organic acids, and epoxy compound production; collectively these compounds are known as secondary oxidation products. The presence of these compounds accounts for the perception of off flavours, rancidity and loss of nutritional value in the food, which can eventually lead to rejection by the customer. Autoxidation of oil has been identified as the main cause of crisps quality deterioration (Choe & Min, 2007) and the reaction rate of autoxidation has been shown to strongly correlate with the shelf-life of the product (Frankel, 1980).

Since the lipid oxidation process occurs at a relatively slow rate at room temperature, it is convenient to use accelerated methods to estimate the oxidative stability and hence the products’ shelf life. Accelerated shelf life tests (ASLT) allow for an evaluation of shelf life in a relatively short time and can be used to accurately predict shelf life for individual products or to compare the storage stabilities of different products, e.g. crisps fried in different oils. It is not straightforward to find the best conditions for ASLT; these conditions depend on the purpose of the study and on the time and facilities available. Many different physical and chemical parameters, such as temperature, metal catalysis, rise in oxygen partial pressure and shaking/forced mixing accelerate oxidation and, as a consequence the development of rancidity. However, since oxidation rates have been shown to increase exponentially with absolute temperature, due to the stability and simplicity of application to control ASLT, this parameter is the most commonly used (Gómez-Alonso et al., 2004, Ragnarsson and Labuza, 1977).

After production, crisps are stored at room temperature, and the initial quality of the product has to be maintained throughout the shelf life of the product. The desire to expand sales in overseas markets and for a more flexible supply chain, has led UK crisps manufacturers to look at methods to extend the shelf life of their products and to withstand temperature fluctuations during transport. Both regular sunflower oil (SO) and high oleic oil sunflower oil (HOSO) provide good frying performances; it has been demonstrated, however, that HOSO is more resistant to oxidative degradation than SO (Niemelä et al., 1996, Sébédio et al., 1996). HOSO has a frying and storage stability comparable to that of more saturated vegetable oils, such as palm olein (Lahtinen et al., 1996, Martin-Polvillo et al., 1996).

Additionally, in recent years, there has been an increase in the use of modified atmosphere packaging (MAP), to further extend shelf life. The three main gases used in MAP are O_2_, CO_2_ and N_2_ and the choice of gas is relative to the food product being packed. Used singly or in combination, these gases are used to balance safe shelf life extension with the optimal organoleptic properties of the food (Bhat, Alias, & Paliyath, 2012). Inert gases have been used in commercial applications for products such as coffee and snack foods, however, the literature on their application and benefits is limited. The most commonly used gas is nitrogen (N_2_).

Potato slices fried by batch processes in kettle fryers have distinctive texture and flavours which are usually recognized by consumers as being distinctly different from those of typical commercially produced continuous processed potato chips. When manufacturing kettle-style fried potato slices, some of the characteristic flavours and texture are believed to be associated with the elevated levels of surface lipids entrapped in the free starch coating, which increases its instability to lipid oxidation. If washed, there is less free surface starch and many of the cooked notes that are associated with fried potato crisps are present at lower levels (Gould, 1999); furthermore crisps with a low surface free starch can be produced with a lower lipid content. Little is known on the shelf life stability of these kettle-style chips (Desai et al., 2014, Johnson, 1990).

To the best of the authors’ knowledge there are no studies comparing the relative effectiveness of HOSO to nitrogen flushing on the storage stability of un-prewashed batch fried crisps. In this study, the relative storage stability of potato slices batch fried industrially in regular SO, in HOSO and in SO subjected to nitrogen gas flushing was compared over an accelerated shelf life storage trial. The primary oxidative degradation products (peroxide value, PV) were measured using a ferrous oxidation-xylenol orange (FOX) assay, while volatile secondary decomposition products (hexanal) were studied by means of solid phase micro-extraction gas chromatography mass spectrometry. This study is unique due to the high free starch on the surface of the sliced fried potato crisps and the combination of approaches evaluated.

## 2 Materials and methods

### 2.1 Potato crisps

Freshly-fried potato crisps were obtained from a commercial batch frying line (Pipers Crisps LTD, United Kingdom), before they were salted and flavoured. The unwashed (high surface starch) potato slices were produced using a batch frying setting. Potatoes were washed prior to slicing in ambient tap water.

### 2.2 Oil and packaging

Crisps were fried in 100% sunflower oil (SO) or 100% high oleic sunflower oil (HOSO) (Kerfoot, Northallerton, United Kingdom). One batch of SO crisps was flushed with nitrogen gas before sealing the packets, with a residual oxygen content of below 2%. The packaging material used was 25 μm OPP Matt Clear/35 μm Met OPP Laminate Film (Roberts Mart, Leeds, United Kingdom).

### 2.3 Reagents and standards

Chloroform, methanol (HPLC grade), dichloromethane (HPLC grade), ethanol (Reagent grade), isopropanol and phenolphthalein were purchased from Fisher Scientific, Loughborough, UK. Xylenol orange tetrasodium salt (ACS reagent grade), cumene hydroperoxide (80%, technical grade), ammonium iron (II) sulphate hexahydrate (ACS reagent grade, 99%), pentanal, heptanal, octanal and nonanal and ethyl butyrate were supplied by Sigma–Aldrich-, Dorset, UK.

### 2.4 Test frying

200 g batches of potato slices were fried in two test fryers (Tefal Easy Pro 2100 W, Ecully Cedex, France) containing 1.8 L SO or HOSO at 175 °C for 300 s, to compare the frying stability of the two oils.

### 2.5 Total polar materials and free fatty acids

Total polar materials (TPM) was measured using a Testo 270 Cooking Oil Tester (Testo, Alton, UK) and free fatty acid (FFA) was quantified by manual titration according to AOCS official method code (AOCS, 1989) with slight modifications: 28.2 g oil taken from a fryer was weighed in a conical flask, 50 mL of propan-2-ol was added, followed by two drops of indicator (phenolphthalein). The solution was mixed and titrated with 0.1 M potassium hydroxide until a stable pink colour was reached. The percentage of FFA in the sample was calculated as follows: FFA% = potassium hydroxide used (mL) × 0.1. Standard error of the methods was <5%.

### 2.6 Accelerated shelf life (ASLT)

Sealed bags of crisps fried in SO, either gas flushed or non-gas flushed, and of crisps fried in HOSO, and were stored at 45 °C. Additional samples were stored at 25 °C and 35 °C for more accurate shelf life predictions (data not shown). Bags were collected at fixed intervals and stored individually in air tight containers at −20 °C prior to analysis.

### 2.7 HS-SPME experimental procedure

#### 2.7.1 Sample preparation

Crisps were ground using a commercial grinder (De Longhi KG49, Havant, UK) at maximum speed for 40 s. Approximately 0.3 g of sample was mixed with 5 mL ultrapure water in 20 mL amber headspace vials (Supelco, Bellefonte, PA, USA) and 100 μL of 1 mmol ethyl butyrate (internal standard) was added. The vials were hermetically capped with PTFE-faced silicone septa (Supelco, Bellefonte, PA, USA). Then, samples were mixed for 30 s in a vortex mixer (Grant, PV1, Shepreth, UK) to form a slurry.

#### 2.7.2 Headspace SPME (HS-SPME)

Analysis was performed with ISQ Single Quadrupole Mass Spectrometer, paired with TRACE 1300 GC, equipped with a ZB-WAX column (30 m × 0.25 mm i.d. × 0.25 μm film thickness), and a TriPlus RSH autosampler (Thermo-Fisher Scientific, Waltham, MA, USA). Following homogenisation, the vials were heated at 70 °C for 5 min to reach equilibrium. A fused silica fibre coated with a 50/30 μm layer of divinylbenzene–carboxen–polydimethylsiloxane (DVD–CAR–PDMS; Supelco) was used to sample analytes from the headspace. Prior to first use, fibres were pre-conditioned, (4 h at 270 °C). After equilibrium, the fibre was exposed to the headspace for a total extraction time of 20 min at 70 °C. After extraction, the fibre was immediately thermally desorbed at 250 °C (5 min).

The oven temperature was as follows: 40 °C for 1 min, then to 120 °C at 20 °C/min, held for 8 min, then to 260 °C at 20 °C/min and finally held for 2 min (Sanches-Silva et al., 2004). MS was operated in electron impact (EI) ionisation mode at 70 eV and data acquisition was achieved at a scan rate of 0.20 s^−1^ over an *m*/*z* range of 35–300. The peak area was processed with Xcalibur Software.

A calibration curve relative was prepared using aqueous standards (25, 50, 100, 150 and 200 ppb), analytes were pentanal, heptanal, octanal and nonanal. Each standard was spiked with 100 μL of ethyl butyrate as an internal standard. Identification of analyte and IS was carried out by comparing GC retention times with previous standard compounds, and confirming the results using NIST MS Search 2.0 library. Relative concentrations were determined using the calibration curve.

### 2.8 Ferrous oxidation-xylenol orange method for lipid hydroperoxide determination

#### 2.8.1 Lipid extraction

Crisps were ground using a commercial grinder (De Longhi KG49) at maximum speed for 40 s. 5 g of ground crisps was transferred into 50 mL centrifuge tubes; 15 mL chloroform/methanol (2:1 vol/vol) was added and the mixture was homogenized on a vortex mixer (Grant, PV1) for 30 s, then centrifuged at 2067*g* for 10 min (Hettich Rotina 380R Newport Pagnell, UK). The bottom layer, consisting of chloroform, was transferred into a fresh 50 mL centrifuge tube and the residue re-extracted twice: first with 15 mL chloroform/methanol (2:1 vol/vol) and then with 5 mL pure chloroform, collecting the lipid rich bottom layer after each extraction and transferring it to the centrifuge tube. The pooled isolates were rinsed with 10 mL (8% wt/vol) aqueous solution of sodium chloride, agitated for 30 s on a vortex mixer (Grant, PV1) and then centrifuged at 2067*g* for 10 min. The chloroform layer (bottom) was transferred into a 28 mL glass vial, gas flushed with nitrogen and stored at −20 °C prior to analysis.

#### 2.8.2 FOX method

The method used as described by Navas and et al. (2004), with slight modifications. The reaction medium consisted of 100 μL 5 mM aqueous ferrous ammonium sulphate, 200 μL 0.25 M methanolic H_2_SO_4_, 200 μL 1 mM methanolic xylenol orange, 1.3 mL dichloromethane/ethanol (3:2, vol/vol), and 200 μL dichloromethane/ethanol (3:2, vol/vol) containing the lipid extract. The chosen final concentration of lipid extract in the reaction medium was 0.5 mg/mL.

Samples were incubated into glass vials with Teflon caps for 30 min, at room temperature and under attenuated light. Absorbance was measured using a spectrophotometer (LBK biochrom, ultrospec 4050) at 560 nm using 1 cm glass cuvettes. Lipid hydroperoxides (LHP) in the samples were quantified using a calibration curve obtained with cumene hydroperoxide in the corresponding reaction medium. The CHP standard solutions used for the calibration curve ranged from 2 to 200 nmol CHP/ml reaction medium. LHP content in samples was expressed as μmol CHP equivalents/g lipid extract.

### 2.9 Statistical analysis

Samples were analysed with three true replicates and statistical analysis was carried out with the Data Analysis tool of Excel. Statistical significance was evaluated with ANOVA and Student’s *t*-test. Statistical differences were considered significant at *P* < 0.05.

## 3 Results and discussion

### 3.1 High oleic vs regular sunflower oil – frying stability

Oxidative stability, and thus the shelf life of the product, depends on a variety of factors, such as heat, light, antioxidants and free metal ions but, in particular, on the fatty acid profile of the oil. Fatty acids, which are in a non-radical singlet state, do not directly react with atmospheric oxygen, which is a di-radical compound (Choe & Min, 2006). Radical oxygen requires that oil be in a radical state for an oxidation reaction to occur; this is achieved by removing a hydrogen atom from the fatty acid chain before reacting with atmospheric oxygen. The energy required to break the carbon—hydrogen bond depends on the position in the fatty acid chain.

Free radical formation at C8 of oleic acid and C11 of linoleic acid requires 75 kcal/mol and 50 kcal/mol respectively, and the overall oxidative rates of oleic acid, linoleic acid and linolenic acid are in the ratio 1:12:25 (Choe, 2007). This means that linoleic acid is less susceptible to oxidation than linolenic acid; however, the most stable is oleic acid. HOSO contains 84% oleic acid, about 60% more oleic acid than regular sunflower oil, and 9% linoleic acid, which is about 54% less than regular sunflower oil (Table 1). Due to its fatty acid profile, HOSO is expected to have better oxidative stability than regular SO (Niemelä et al., 1996) which supports the data herein.

### 3.2 Determination of lipid hydroperoxides in crisps using the FOX method

**Fig. 1:**
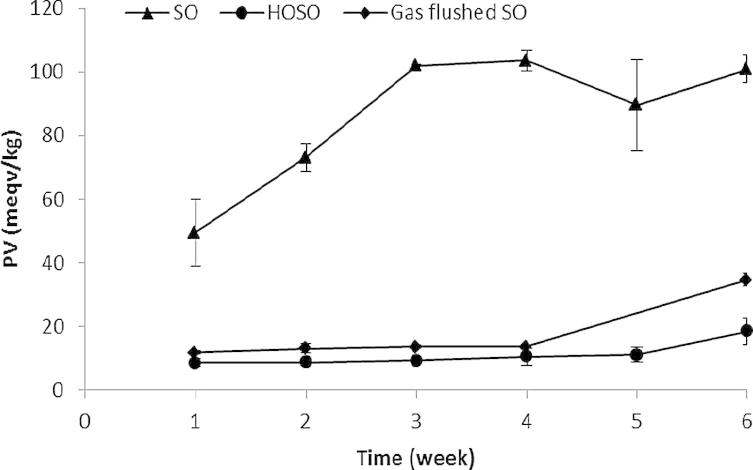
Peroxide value (meqv/kg) in nitrogen gas flushed crisps compared with non-gas flushed crisps fried in SO and HOSO during 6 weeks storage at 45 °C. Due to storage facility failure, data relative to gas flushed SO crisps at week 5 were not available.

There was a significant increase (*P* < 0.05) in PV for SO, HOSO and gas flushed SO crisps over the course of the storage trial (Fig. 1). At week 1, crisps fried in SO had a PV of 49.5 meqv/kg whereas those fried in HOSO had a PV of 8.4 meqv/kg. The PV of SO crisps also increased at a faster rate than that of HOSO crisps, and reached 101 meqv/kg by week 6 whereas the PV of HOSO crisps increased by only 18.5 meqv/kg over 6 weeks. The finding that HOSO fried crisps are more stable than SO fried crisps is in accordance with results published previously (Lahtinen et al., 1996, Navas et al., 2004).

Gas flushed crisps were more stable than regular SO crisps with a PV of 13 meqv/kg at the beginning of the storage trial (weeks 1–4), although nitrogen flushed crisps were not as stable as HOSO crisps. At the end of the storage trial, the PV of gas flushed crisps was 34.5 meqv/kg, which significantly exceeded the peroxide value of HOSO crisps, and was significantly lower than that of SO crisps (*P* < 0.05).

### 3.3 Measurement of volatile compounds in crisps using the Headspace SPME (HS-SPME) method

Several volatile compounds were found in the potato crisps, in particular the saturated aldehydes pentanal, hexanal, heptanal, octanal and nonanal as these are all commonly recognised markers for secondary oxidation.

Hexanal was chosen as the primary indicator of secondary oxidation as it has previously been reported as being a good indicator of lipid oxidation in potato crisps (Azarbad, 2014, Jeon, 1984, Sanches-Silva et al., 2004).

**Fig. 2:**
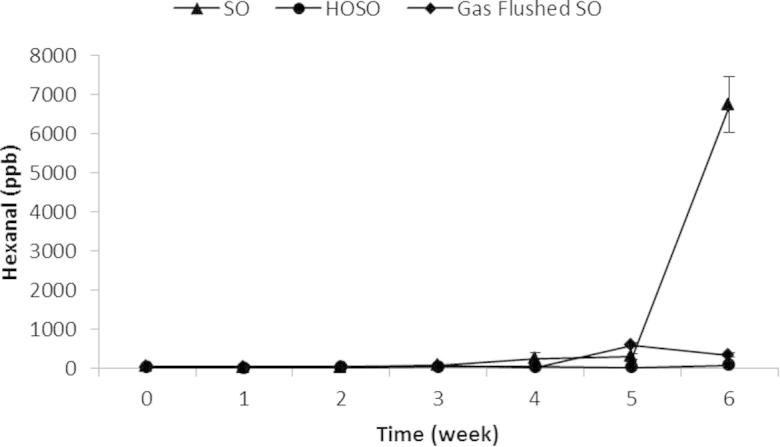
Hexanal concentration (ppb headspace) in nitrogen gas flushed crisps compared with non-gas flushed crisps fried in SO and HOSO during 6 weeks storage at 45 °C.

Headspace hexanal concentration in SO crisps increased significantly at week 6, while the concentration in HOSO crisps did not increase significantly over the course of the study (*P* < 0.05) (Fig. 2). SO crisps were stable for the first three weeks, with hexanal values below 100 ppb equivalent. During weeks 3 to week 5, oxidation starts to occur and after week 5 there was a rapid production of secondary oxidation products, with headspace hexanal concentrations reaching 6700 ppb at week 6 for SO crisps, whereas HOSO crisps did not exceed 110 ppb at any time during the study. The results obtained here for the accelerated shelf life are in agreement with those shown by (Lahtinen et al., 1996, Martin-Polvillo et al., 1996).

Autoxidation of oils and the decomposition of hydroperoxides has been shown to increase as temperatures increase (St Angelo, 1996). A simple way to express this acceleration is to use the Q_10_ concept. Q_10_ is the increase in the rate of a reaction (R) when the temperature (T) is increased by 10 °C.

$$Q_{10}={\left(\frac{R_{1}}{R_{2}}\right)}^{\left(\frac{10}{T_{2}-T_{1}}\right)}$$

For the purpose of this study an approximated Q_10_ value of 2 was used, meaning that the rate of oxidation doubled when storage temperature increased by 10 °C (Frankel, 1980). According to this approximation, 1 week at 45 °C corresponded to 2 weeks shelf life at 35 °C and 4 weeks at ambient temperature (25 °C). Due to the low increase in PV and hexanal in the accelerated shelf life test, HOSO crisps were predicted to be potentially stable for up to 24 weeks (6 months real shelf life).

The impact of gas flushing on lipid oxidation was also evaluated and the results are shown in Fig. 2. While in SO fried crisps hexanal concentration increased significantly (*P* < 0.05) and reached 6700 ppm at week 6, in gas-flushed crisps hexanal concentrations only reached 590 ppb (weeks 5 and 6) supporting the hypothesis that nitrogen gas can dramatically reduce the oxidation progression in unwashed fried sliced potato crisps (*P* < 0.05).

Despite the effectiveness of gas flushing, crisps fried in HOSO were more stable than SO or SO gas flushed crisps concluding that of the tested approaches, HOSO is the most appropriate oil for crisp production of this type (Fig. 2).

The data presented is in accordance with sensory assessments of stored French fries and crisps fried in SO and HOSO carried out previously by Raoux et al., 1996, Van Gemert, 1996 which revealed that crisps fried in HOSO had a shelf life that was very similar to crisps fried in the very stable palm oil. The “desired” fruity characters (odour and taste) were stable for up to week 27 for HOSO, while crisps fried in SO were rancid at week 17 (weeks 4/5 in accelerated shelf life). This finding confirmed the higher oxidative stability of HOSO when compared to SO.

**Fig. 3:**
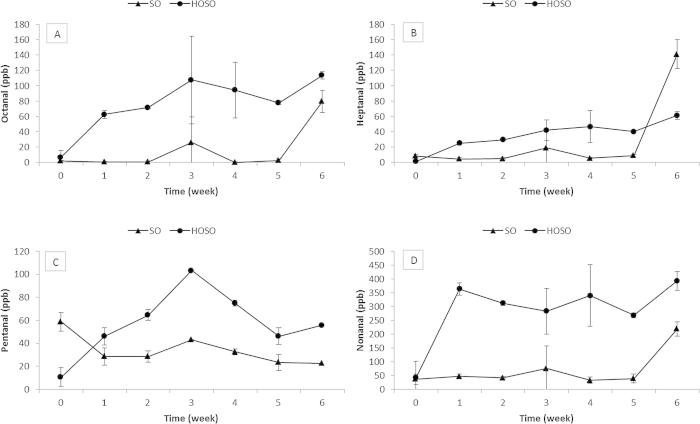
Aldehydes detected in the samples during the storage trial at 45 °C for 6 weeks, in SO and HOSO: (A) pentanal, (B) heptanal, (C) octanal, (D) nonanal.

The concentrations of other aldehydes, namely pentanal, heptanal, octanal and nonanal during the storage trial were determined (Fig. 3). Results revealed that octanal and nonanal are higher in HOSO then in SO (Fig. 3C and D). This is due to the higher oleic acid content of HOSO then SO. Octanal and nonanal are produced preferentially from 11 and 10 oleic acid hydroperoxides breakdown (Nuchi, Julian McClements, & Decker, 2001). It should be noted that the relative abundance of all of the non-hexanal markers was very low compared to hexanal but that they all tended to increase towards the end of the study.

### 3.4 Frying stability over multiple frying cycles

**Fig. 4:**
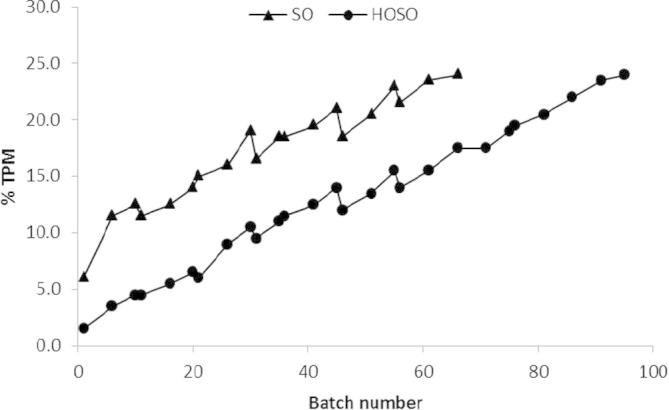
Change in total polar molecules (% TPM) in SO and HOSO over multiple frying cycles.

**Fig. 5:**
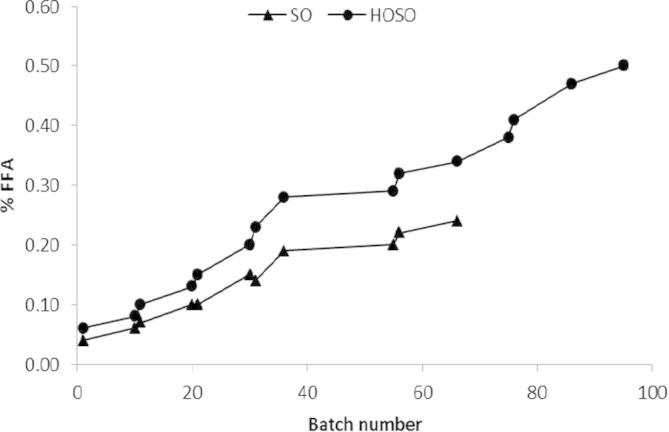
% free fatty acids (FFA) in SO and HOSO over multiple frying cycles.

Total Polar Materials (TPM) are non-volatile oil degradation products formed during frying, such as dimers and oligomers, TPM is often considered as one of the best indicators of oil frying life; as a measure it is more reliable and comprehensive than free fatty acids an alternative marker for stability (Oreopoulou, Krokida, & Marinos-Kouris, 2006). The frying stability of SO and HOSO was measured over multiple frying cycles, SO fried crisps reached 24% TPM after frying 66 frying cycles, however HOSO was more stable and did not reach this level until 95 frying cycles. This finding further demonstrates an enhanced stability to oxidative deterioration (Fig. 4, Fig. 5).

Prior studies have also shown that TPM increases over multiple frying cycles (Houhoula et al., 2002, Richard et al., 1993). While there is no legal limit in the UK, EU regulations suggest that when oil reaches 24% TPM, its frying life should be terminated and the oil should be discarded (Firestone, Stier, & Blumenthal, 1991). This limit may be used as a guideline for UK crisp manufacturers.

FFA increased over multiple frying cycles at a slower rate in SO when compared to HOSO (Fig. 5). However, after 66 batches, despite FFA being 0.24% SO was discarded due to the high TPM concentrations (Fig. 5) FFAs are produced during frying through reaction with moisture (hydrolysis) and, in a similar mechanism to TPM, their concentration increases as food is fried. There is no EU regulation for FFA but their concentration should be maintained as low as possible (typically below 0.5%) due to the possible impact on taste (Chalé-Rush et al., 2007, Fritch, 1981, Mattes, 2009). Higher levels of FFA have been shown to adversely affect shelf life (Kamal-Eldin, 2006). Among UK crisps manufacturing companies, 0.5% FFA is commonly considered the maximum limit of acceptability.

## 4 Conclusions

In summary, exchanging SO for HOSO resulted in a significant increase in predicted shelf life of unwashed fried potato crisps. Unwashed crisps stabilised by nitrogen gas flushing also performed well, but were not as stable as the crisps fried in HOSO. HOSO was also more stable during multiple frying cycles when compared to SO (TPM). Therefore, in addition to fresh HOSO frying oil extending the shelf life of unwashed fried potato crisps, HOSO also resulted in an extended frying stability. Depending on the shelf life requirements, the optimal commercial solution could be a combination of intelligent frying oil choice, oxidation limit selection and use of nitrogen gas flushing to ensure unwashed (high free starch) fried sliced potatoes crisps are sufficiently stable.

## Figures and Tables

**Table 1:** Fatty acid composition of common vegetable oils. Trace = below the limit of detection.

	Fatty acid composition (%)				
Palmitic (C: 16)	Stearic (C: 18)	Oleic (C18:1)	Linoleic (C18: 2)	Alpha linolenic (C18: 3)
SO	7	4	24	63	Traces
HOSO	4.5	3.5	84	9	Traces
Palm oil	45	5	38	10	Traces
Olive oil	14	3	71	10	Traces
